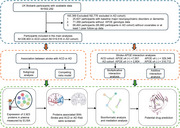# *APOE* Genotype modulate the relationship of stroke with dementia risk: associations and peripheral biological mechanisms based on a large prospective cohort and blood proteomics investigation

**DOI:** 10.1002/alz70861_108163

**Published:** 2025-12-23

**Authors:** Wei Xu, Liangyu Huang, Lan Tan

**Affiliations:** ^1^ Qingdao Municipal Hospital, Qingdao University, Qingdao, Shandong China; ^2^ Qingdao Municipal Hospital, Qingdao, 266071 China; ^3^ Qingdao Municipal hospital, Qingdao university, Qingdao, Shandong China

## Abstract

**Background:**

Stroke and *APOE* ε4 are established risk factors of dementia and Alzheimer’s Disease (AD). However, it remains unclear whether stroke interacts with *APOE* genotypes to influence dementia occurrence. This study aims to investigate the associations of stroke and its interaction by *APOE* genotypes with incident risk of dementia, with a specific focus on underlying peripheral biological mechanisms.

**Methods:**

This prospective cohort study included 336,903 participants (mean age: 56.3 years, stroke history: 1.3%, *APOE* ε4: 28.5%) from the UK Biobank, with a median follow‐up of 13 years. The death‐competing risk models based on Cox proportional hazards regression were employed to examine the additive and multiplicative interaction of stroke and its subtypes with *APOE* genotypes on incident risk of all‐cause dementia (ACD) and AD. Blood proteomics combined with bioinformatics analyses were used to explore the interplay mechanisms.

**Results:**

Either ischemic or hemorrhagic stroke was significantly associated with elevated risk of ACD and AD (P < 0.001). A significant multiplicative interaction was observed between stroke and *APOE* ε4 (P < 0.001). The association of stroke with increased risk of dementia was stronger in *APOE* ε4 non‐carriers than carriers, for both ACD (hazard ratio [HR] = 1.93 for carriers and 3.36 for non‐carriers, *p* < 0.001) and AD (HR = 1.14 for carriers and 2.67 for non‐carriers, *p* < 0.001). Cell surface receptor signaling pathways, regulation of multicellular biological processes, and cytokine‐cytokine receptor interaction pathways could be mechanisms underpinning the association of stroke with ACD risk. We identified 191 functionally interconnected (P < 1.0 × 10^‐16^) proteins associated with both stroke and ACD only in *APOE* ε4 non‐carriers. CD4‐related and TGF‐beta signaling pathway could mediate the strengthened relationship in *APOE* ε4 non‐carriers.

**Conclusions:**

Stroke interacts with *APOE* ε4 to influence all‐cause or Alzheimer’s dementia, with the association being more pronounced in *APOE* ε4 non‐carriers. Future studies are needed to verify the underpinning mechanisms to guide precise prevention.